# Differential Outcomes of Adolescents with Chronically Ill and Healthy Parents

**DOI:** 10.1007/s10826-012-9570-8

**Published:** 2012-02-22

**Authors:** Dominik Sebastian Sieh, Johanna Maria Augusta Visser-Meily, Anne Marie Meijer

**Affiliations:** 1 Research Institute of Child Development and Education, University of Amsterdam, Nieuwe Prinsengracht 130, 1018 VZ Amsterdam, The Netherlands; 2 Rudolf Magnus Institute of Neuroscience, University Medical Center Utrecht, Heidelberglaan 100, 3584 CX Utrecht, The Netherlands

**Keywords:** Parental chronic medical condition, Adolescent, Adjustment, Family

## Abstract

Approximately 10% of children grow up with a parent who has been diagnosed with a chronic medical condition (CMC) and seem to be at risk for adjustment difficulties. We examined differences in behavioral, psychosocial and academic outcomes between 161 adolescents from 101 families with a chronically ill parent and 112 adolescents from 68 families with healthy parents, accounting for statistical dependence within siblings. Children between 10 and 20 years and their parents were visited at home and filled in questionnaires. Multilevel analyses showed that 20–60% of the variance in most adolescent outcomes was due to the family cluster effect, especially in internalizing problem behavior, caregiving variables and quality of parent attachment. Conversely, the variance in stress and coping variables and grade point average (GPA) was mainly due to individual characteristics. Adolescents with parents affected by CMC displayed more internalizing problems than the comparison group and scored higher on frequency of household chores, caregiving responsibilities, activity restrictions, isolation, daily hassles and stress. In addition, their grade point average was comparatively worse. No group differences in externalizing problems, coping skills and quality of parent attachment were found. In conclusion, the family cluster effect largely explains adolescent outcomes and should be accounted for. Adolescents with parents affected by CMC are subject to an increased risk for internalizing problems, adverse caregiving characteristics, daily hassles, stress and a low GPA. According to a family-centered approach, school counselors and health care practitioners should be alert to adjustment difficulties of children with a chronically ill parent.

## Introduction

Approximately 10% of children have a parent with a chronic medical condition (CMC) and seem to be at an increased risk for persistent stress and adjustment difficulties (Sieh et al. 2010a, b; Verhaeghe et al. 2005; Visser-Meily et al. 2005). A meta-analysis of 19 studies including a total of 1,858 children of parents affected by CMC revealed that they displayed more internalizing problems such as anxious, depressed and withdrawn behavior and somatic complaints than children with healthy parents or norm groups (Sieh et al. 2010a).

Generally, the body of research on CMC includes illness-specific studies and studies using mixed illness samples and or/self-identified caregivers. The majority of studies focusing on specific diagnoses are cancer studies. Children of parents with cancer show less problem behavior than children in studies investigating other CMC’s than cancer (Sieh et al. 2010a). However, several studies still report particular problems. For instance, a study concluded that children (*N* = 27) of cancer patients perceived their risk of developing cancer as significantly higher than controls (Harris and Zakowski 2003). A study of more than 200 adolescents revealed elevated stress symptoms in 21% of boys and 35% of girls (Huizinga et al. 2005b). In addition, these adolescents appeared to communicate less openly with their parents (Huizinga et al. 2005a). A review of 52 studies showed that children displayed signs of anxiety and depression in both qualitative and quantitative research (Visser et al. 2005).

The remaining illness-specific studies have mainly focused on HIV and/or hemophilia, multiple sclerosis, Parkinson disease, rheumatoid conditions and stroke. Children whose mothers were infected with HIV developed more externalizing problems than children whose mothers were not infected (Tompkins and Wyatt 2008). A large study of more than 300 children of parents infected with HIV showed that these children had more life stressors, family conflict and lower self-esteem than controls (Wu et al. 2008). Forehand et al. (1998) compared 87 African American children of parents infected with HIV to controls, stating that children of parents with HIV had more difficulties in all domains of psychosocial adjustment including problem behavior and reading achievement scores. Diareme et al. (2006) found that children of mothers with multiple sclerosis exhibited increased scores on domains of emotional and behavioral problems in comparison with controls. Children of parents with Parkinson disease (*N* = 77) reported a high frequency of daily hassles affecting their personal life (Dufour et al. 2006). Evans et al. (2007) examined attachment in children of mothers with chronic pain resulting from arthritis, other conditions or no medical condition, concluding that these children had more insecure attachment than controls. Further, children of parents with stroke frequently exhibited daily hassles (Dufour et al. 2006) and elevated levels of problem behavior even several years post-stroke (Visser-Meily et al. 2005).

Studies with mixed illness samples and/or studies of self-identified caregivers are often retrospective or qualitative of nature. From qualitative research, it can be concluded that anxious symptoms are especially pronounced and oftentimes constitute worries about health-related issues (e.g., Dearden and Becker 2000). Banks et al. (2002) deducted from their interviews of self-identified carers that academic achievement was relatively poor, explaining that young caregivers frequently leave school right after the minimum leaving age. Two quantitative studies have focused on self-identified caregivers of chronically ill parents. A quantitative study on hundred self-identified caregivers aged ten to 25 years found that caregiving and family responsibilities were highly common. In addition, young caregivers scored lower on life satisfaction than non-caregivers. Their reliance on problem solving and social skills was comparatively low (Pakenham and Bursnall 2006; Pakenham et al. 2006). Houck et al. (2007) administered quantitative measures to 38 adolescents and found clinical levels of posttraumatic symptoms in one-third of the sample.

In conclusion, children with a chronically ill parent seem to have more adverse outcomes in behavioral, psychosocial and academic adjustment than other children. Although the effects are small across studies, it should not be neglected that adjustment difficulties may pose a threat to a healthy development of these children.

This study responds to several boundaries of previous research. First, researchers have not always corrected for the statistical dependence between members from the same family, leading to violation of the assumption of independence. To our knowledge, only Visser-Meily et al. (2005) have examined the clustering structure of families with a chronically ill parent, using multilevel analysis. Families share the same environment and children from the same family may therefore score similarly on outcome variables (Tabachnik and Fidell 2007). Consequently, data of siblings within families require researchers to be considerate of the between-subject dependence. The family cluster effect is related to multiple factors of genetics and environment. Second, many studies in the field omitted comparison groups and therefore lack the possibility to draw conclusions about specific characteristics and needs of children with a chronically ill parent. In addition, those studies including a comparison group mostly focused on problem behavior as measured with the Child Behavior Checklist or the Youth Self Report (Sieh et al. 2010a). As far as we know, only Pakenham et al. (2006) have compared specific characteristics (e.g., caregiving context variables) of children with a chronically ill parent compared to other children. However, this sample is composed by caregivers and non-caregivers with a wide age range. Instead, we focus on adolescents with a chronically ill parent (target group) because they appear to be a particularly vulnerable group (Kraaij et al. 2003; Visser et al. 2005). To our knowledge, no study has treated statistical dependencies between adolescents from the same families, using a mixed illness sample. It is therefore largely unknown on which variables and to what extent teenagers in families with a chronically ill parent differ from teenagers with healthy parents in regard of the strong between-subject dependence in families.

Our first aim is to examine whether adolescent outcomes are rather explained by the family cluster effect than by individual characteristics. Our second aim is to identify group differences in a wide variety of outcome variables by comparing the target group with controls. Following the meta-analysis by Sieh et al. (2010a), we examine internalizing and externalizing problem behavior. In addition, this study compares caregiving variables and stress and coping variables that may constitute specific characteristics of the target group. Further, we investigate parent attachment and academic achievement (e.g., grade point average) because these variables are understudied. Thus, this study analyzes problem behavior, caregiving characteristics, daily hassles, stress and coping behavior, quality of parent attachment and grade point average. We hypothesize that the target group displays more problem behavior and scores higher on caregiving variables (i.e., frequency of household chores, caregiving responsibilities, activity restrictions and feeling of isolation) than controls. We also presume that the target group reports more daily hassles, more global psychological stress and lower levels of active problem solving and social support seeking than controls. Finally, we suppose that the quality of parent attachment is lower and that the grade point average is worse in the target group compared to controls.

## Method

### Participants and Procedure

Children aged 10–20 years living at home and their parents were included. Children in the target group had to have a parent with one or more CMC’s for at least 6 months. Cancer was excluded because oncological disease is not chronic by definition. Children from the comparison group had to have two parents with no major somatic illness. Exclusion criteria for adolescents were insufficient command of Dutch, residency outside of the Netherlands, severe somatic diseases and cognitive disabilities. Having a light somatic disease like asthma was not an exclusion criterion for participants.

Participants were recruited across the Netherlands in 5 rehabilitation centers and several hospitals, schools, community centers, intercultural institutions, general health practitioners’ offices and public libraries across the country between September 2008 and April 2010. Apart from distributing posters and brochures, professionals of the participating institutions provided additional oral information about this study and invited potential families to participate. Besides, information was posted on the websites of patient organizations of the diagnostic groups included in the study. Potential participants (both parents and children) could contact the project manager by mail or phone to receive more information and make a request to receive an informed consent form. After written informed consent had been given by parents and children, several trained research assistants made an appointment to administer questionnaires at the families’ homes. Children from the target and control group filled in a test battery covering the outcome variables and both of their parents filled in a short questionnaire measuring demographic variables and illness characteristics. The research assistants followed a research protocol which had been designed and trained by the project manager. Adolescents could choose between a gift voucher, a cinema ticket or a mobile phone cover after completing the questionnaires. The participating families received information about the status of the research project at 4 occasions through a newsletter. The study was approved by the ethical commission of the research institute of Child Development and Education of the University of Amsterdam.

Six participants in the target group who contacted us did not meet the inclusion criteria and 6 participants decided not to participate, yielding a total of 161 adolescents from 101 families. Of these adolescents, 99.4% was Caucasian and .6% was Surinamese. Controls were 112 adolescents with two healthy parents from 68 families. The ethnic composition of the comparison group was 96% Caucasian, 2% Surinamese, 1% Indonesian and 1% Yemeni.

### Measures

#### Demographic Variables

Questionnaires for parents and children included questions about gender, age, employment status and educational level. The questionnaire for parents with CMC additionally included questions about illness type and duration and family income.

#### Problem Behavior

Internalizing and externalizing problem behavior in adolescents was measured with the Youth Self Report (Achenbach 1991). Adolescents rated their behavioral problems on a 3-point scale as *not true* (0), *somewhat/sometimes true* (1) or *very/often true* (2). Items were summed to obtain a total score for internalizing symptoms (31 items, Cronbach’s alpha (α) = .88) and externalizing symptoms (30 items, α = .73). Cronbach’s alpha’s for the internalizing symptom subscales *withdrawn behavior*, *somatic complains* and *anxiety/depression* showed satisfactory to good reliability (α = .65, α = .71 and α = .86). This was also true for the externalizing symptom subscales *aggressive behavior* (α = .60) and *rule breaking behavior* (α = .79).

#### Caregiving Variables

All items measuring caregiving variables were unrelated to parental illness because caregiving could not have been compared to controls otherwise. Adolescents filled in three subscales of the Young Caregiver of Parent Inventory (YCOPI) from Pakenham et al. (2006). These scales were (back)translated by a bilingual speaker to create the Dutch version. We only used three scales of the YCOPI because Dufour et al. (2006) and Meijer et al. (2008) designed several questionnaires that emerged as a suitable and valid alternative for the Dutch population. The three Dutch scales of the YCOPI were *caregiving responsibilities* (8 items, α = .77), *activity restrictions* (8 items, α = .85) and *feeling of isolation* (3 items, α = .74). Examples for these scales were *Others expect me to help my parents.*, *I feel as though I am missing out on things.* and *I sometimes feel alone*, respectively. Adolescents rated the extent to which they agreed on each item, using a 5-point scale ranging from *strongly disagree* (0) to *strongly agree* (4). In addition, they filled in the subscale *frequency of household chores* (α = .64) of the Dutch Caregiving Inventory. Household chores related to items like cleaning the house and putting out garbage. Higher scores indicated more household chores as reported by adolescents. The scale contained 8 items and was answered on a 5-point scale (i.e., *not at all*, *less than once a week*, *1*–*3 times a week*, *3*–*6 times a week* and *daily*) (Meijer et al. 2008).

#### Stress and Coping Variables

We measured how often children perceived stressful events in their environment that had impact on their personal life, using the subscale *frequency of daily hassles affecting personal life* of the Daily Hassles Questionnaire (Dufour et al. 2006). Personal life referred to social time with friends, school duties and the possibility of having a job (e.g., *How often does your family situation affect your homework?*). From the original 8-item version, 2 items had to be discarded because they included an illness-related matter. The Daily Hassles Questionnaire had the same scoring as the Dutch Caregiving Inventory and showed satisfactory reliability (6 items, α = .65). We further administered the Dutch Stress Questionnaire for Children*,* a 17-item child-report measure assessing psychological stress (α = .85). Scores ranged from 17 to 68; a higher score indicated more stress (Hartong et al. 2003). Coping behavior was measured with two 6-item scales of the Utrecht Coping List for Adolescents: *seeking social support* (α = .88) and *active problem solving* (α = .79). We only used these two scales because the validation study showed that the other scales had comparatively low reliability (Schreurs et al. 1993).

#### Quality of Parent Attachment

Adolescents answered 12 items about the attachment with fathers (α = .87) and mothers (α = .85) separately, evaluating the parent attachment. Items were deducted from the Inventory of Parent and Peer Attachment and were a sum of the subscales *communication*, *confidence* and *alienation* which were rated on a 4-point scale from *almost never* (1) to *almost always* (4) (Armsden and Greenberg 1987). Higher scores indicated higher quality of attachment with the father or mother.

#### Academic Achievement

Adolescents reported their grade point average (GPA) of the previous scholar year on a scale from 4 or lower (insufficient) to 10 (excellent). In addition, we calculated the percentage of children who had ever failed a school year.

### Statistical Analysis

In regard of the variances and distributions of the scores, we used descriptive statistics, one-way ANOVA’s, Chi square tests and Mann–Whitney tests to describe group characteristics and group differences. We conducted multilevel analyses (MLA) to test group differences and to take dependencies between subjects coming from the same families into account, using dummies for the target group (1) and controls (0). MLA is a type of regression analysis suitable for data with a nesting structure (children within families), correcting for the violation of the assumption of independence (Snijders and Bosker 1999). The intraclass correlation coefficient (ICC) was calculated to examine how much variance in adolescent outcomes was explained by the family cluster effect.

We calculated the effect size *Cohen’s* *d* as a function of means and standard deviations of the outcome scores. As a rule of thumb, effect sizes of *d* = .30, *d* = .50 and *d* = .80 can be considered small, medium and large, respectively (Cohen 1992). In this study, a family had 1.6 children and the ICC for the outcome variables was .33 on average, resulting in a design effect of 1.20. The power to detect medium effects (power = .80, significance = .01) required 225 cases. Our sample consisted of 273 cases, so we had enough power to detect medium effects.

Missing data only applied to 5% of cases at most and could be viewed as random sample of the cases, which is why the data were handled through Expectation Maximization (Graham 2009). All significance tests were two-tailed.

## Results

### Description of Illness Characteristics

Of the parents with CMC, 67% was female. Parental CMC included multiple sclerosis (28.2%), rheumatoid arthritis (19.4%), brain damage (16.5%), muscle disease (14.6%), spinal cord injury (6.8%), inflammatory bowel disease (5.8%), Parkinson disease (5.8%), and diabetes type I with physical complications (2.9%). The mean time since diagnosis was 12.4 years and ranged between 7 months and 49 years. Prior analyses revealed that illness duration and illness characteristics were not strongly related to adolescent outcomes, so we did not categorize the target group into subgroups based on illness characteristics (Sieh et al. 2011).

### Demographic Group Differences of Parents

Descriptive analyses illustrated that parents’ age and education did not differ between the groups, see Table 1. Withal, only 36% of parents with CMC was employed in comparison to more than 90% of parents from the comparison group. Healthy partners from the target group compensated for this discrepancy, lifting the average employment rate to more than 60%. The Mann–Whitney test revealed that parents from the target group earned significantly less money than controls (*z* = −4.02, *p* < .001). Parents who were employed had close to 30 work hours per week in both groups. Almost two-thirds of parents with CMC received financial aid from the government, whereas the comparison group hardly received any financial support. Almost every second family including a parent with CMC received less income after the diagnosis. Per month, the financial deterioration was 680 Euro’s after taxes.

**Table 1:** Demographics of parents from the target and comparison group

	Target group (*N* = 187)	Comparison group (*N* = 136)
Mean age (*SD*)	47.1 (5.5)	47.7 (5.1)
Currently employed	63.5%	91.1%
Mean work hours per week (*SD*)	29.9 (14.1)	31.4 (12.1)
Mean net family income per month in Euro’s (*SD*)	2700 (965)	3190 (868)
Financial aid from the government	37.9%	.7%
School type		
Mean education^a^ (*SD*)	4.1 (1.4)	4.4 (1.3)
Primary/lower education	13.7%	7.4%
Intermediate vocational education	29.6%	28.1%
High school	9.5%	7.4%
(Pre-)university education	46.6%	55.6%

^a^Education level ranges from 1 primary education to 5 = (Pre-)university education

### Demographic Group Differences of Adolescents

Twelve percent of the target group and 8% of the comparison group indicated to have a light somatic illness themselves. The illnesses reported by adolescents were asthma (54%), followed by post-Pfeiffer symptoms (14%) and other light somatic conditions (32%) such as hypothyroidism.

Fifteen percent of the target group had a single parent who was chronically ill. The groups of adolescents did not differ in terms of gender, age, mean educational level and illness status (*p* > .05), see Table 2. Notably, the target group followed intermediate vocational education more than twice as often as controls. On the contrary, for every 10 controls, 6 children of parents with CMC followed high school. Moreover, in both groups, approximately 4 out of 10 participants had a paid job next to school. The target group worked slightly more hours per week than the comparison group (*b* = 1.56, *p* = .05).

**Table 2:** Demographics of adolescents from the target and comparison group

	Target group (*N* = 161)	Comparison group (*N* = 112)
Female	51.6%	53.6%
Mean age (*SD*)	15.1 (2.3)	15.0 (2.4)
Ratio of participating adolescents per family	1.59	1.65
Mean number of children per family (*SD*)	2.0 (.99)	2.0 (.97)
Healthy^a^	88.2%	92.0%
School type		
Mean education^b^ (*SD*)	2.5 (1.1)	2.5 (.9)
Primary education	16.9%	12.6%
Lower vocational education	39.4%	35.1%
Intermediate vocational education	14.3%	6.4%
High school	25.0%	42.3%
(Pre-)university education	4.4%	3.6%
Having failed at least one school year	18.0%	15.2%
Having a job	42.2%	43.8%

^a^Healthy refers to absence of light somatic disease

^b^Education level ranges from 1 primary education to 5 = (Pre)university education

### Multilevel Analyses: The Family Cluster Effect

Multilevel analyses were conducted to account for the nesting structure of adolescents (Level 1) within families (Level 2), meaning that we controlled for unobserved differences between families and avoided an overestimation of group differences (Snijders and Bosker 1999). The ICC’s revealed that approximately 41% and 24% of the variability in internalizing and externalizing problems, respectively, was due to family membership, meaning that the family cluster effect explained the variance in adolescent internalizing problems almost twice as strongly as externalizing problems, see Table 3. Caregiving characteristics had high ICC’s, meaning that the family cluster effect contributed majorly to adolescent engagement in caregiving-related activities. To illustrate, the majority of variability in frequency of household chores was explained by the family-clustering effect (ρ = .59). The variance of other factors that was highly influenced by the family cluster effect were quality of attachment with the father (ρ = .53) and quality of attachment with the mother (ρ = .39). On the contrary, the ICC’s for coping behavior, daily hassles, stress and GPA were moderate, demonstrating that the main part of adolescents’ scores was explained by individual factors rather than the family cluster effect. Active problem solving proved to be the only factor that was almost entirely explained by individual characteristics.

**Table 3:** Differences between the target group and controls in problem behavior, caregiving variables, stress and coping variables, quality of attachment and grade point average using multilevel analyses

	ICC	Target group *M* (*SD*)	Comparison group *M* (*SD*)	Estimate	Effect size
*Problem behavior*					
Internalizing problems	.41	9.71 (8.66)	7.46 (4.97)	2.33**	.31
Depressed/anxious behavior	.34	4.64 (5.14)	3.29 (3.06)	1.32**	.31
Withdrawn behavior	.45	2.40 (2.28)	1.90 (1.74)	.47	.24
Somatic complaints	.23	2.90 (2.93)	2.41 (2.08)	.60*	.19
Externalizing problems	.24	7.52 (5.43)	7.17 (4.92)	.15	.07
Aggressive behavior	.16	4.83 (3.82)	4.75 (3.61)	.02	.02
Delinquent behavior	.31	2.65 (2.50)	2.42 (2.09)	.14	.10
*Caregiving variables*					
Caregiving responsibilities	.33	12.52 (5.68)	11.01 (4.81)	1.48**	.28
Activity restrictions	.45	5.63 (5.46)	3.47 (3.59)	2.09***	.45
Feeling of isolation	.40	3.73 (3.07)	2.77 (2.13)	.89**	.35
Frequency of household chores	.59	6.70 (3.65)	4.65 (2.70)	1.96***	.62
*Stress and coping variables*					
Frequency of daily hassles	.29	2.19 (2.97)	1.50 (1.79)	.73**	.27
Stress	.23	34.78 (8.21)	32.87 (6.30)	1.82*	.26
Active problem solving	.01	14.35 (3.44)	14.39 (3.44)	−.04	−.01
Social support seeking	.28	13.68 (4.23)	13.77 (3.90)	−.11	−.02
*Quality of attachment*					
Quality of attachment with father	.56	37.07 (7.28)	37.98 (5.33)	−.66	−.14
Quality of attachment with mother	.39	40.60 (6.01)	41.05 (4.74)	−.38	−.08
*Grade point average* ^a,b^	.27	6.92 (.87)	7.28 (.76)	−.35***	−.44

*ICC* intraclass coefficient. ICC’s indicate how much variance in adolescent outcomes was explained by family environment. All scores presented are raw scores. Effect sizes are Cohen’s *d*

* *p* < .10, ** *p* < .05, *** *p* < .01

^a^Grades range from 1 = very poor to 10 = excellent

^b^Due to non-normal distribution and scale properties, the Mann–Whitney test was used for this variable

### Differential Outcomes in Behavioral, Psychosocial and Academic Domains

In consideration of the strong statistical dependencies within families, a high number of significant group differences emerged (Table 3). Children of parents with CMC scored higher on internalizing problem behavior (*b* = 2.33, *p* = .03) than children of healthy parents. The difference in internalizing problems was largely attributable to differences in anxious-depressive symptoms (*b* = 1.32, *p* = .03). The group difference in somatic complaints was only significant with a .10 significance level (*b* = .60, *p* = .09), while the difference in withdrawn behavior was not significant. Similarly, the groups did not differ in externalizing problems. In comparison with controls, the target group scored higher on frequency of household chores (*b* = 1.96, *p* = .00), caregiving responsibilities (*b* = 1.48, *p* = .05), feeling of isolation (*b* = .89, *p* = .02) and activity restrictions (*b* = 2.09, *p* = .00). The target group also scored comparatively higher on frequency of daily hassles affecting personal life (*b* = .73, *p* = .04) and slightly higher on stress (*b* = 1.82, *p* = .07). Finally, the target group had a worse GPA than controls (*b* = −.35, *p* = .00). In addition, the percentage of the target group who failed a school year was slightly higher in comparison with controls. No group differences were found on coping skills and quality of parent attachment. Significant effect sizes were generally small, but for caregiving variables and GPA, the effect sizes were medium.

## Discussion

This study is, to the best of our knowledge, the first to assess key differences between adolescents with a chronically ill parent and adolescents with healthy parents on a wide array of behavioral, psychosocial and academic outcomes, using a multilevel design that accounts for the family cluster effect. The results show that the target group scored higher on internalizing problem behavior, caregiving variables, daily hassles and stress and lower on GPA than controls. The family cluster effect had a major contribution to the variance of many adolescent outcome variables such as internalizing problem behavior, caregiving variables and quality of parent attachment. Contrarily, the variance of stress and coping variables was mainly due to adolescents’ individual characteristics. Active problem solving coping was entirely explained by individual characteristics.

Regarding problem behavior, the target group reported more anxious and depressive behavior and slightly more somatic complaints in comparison with controls, but the groups did not differ in externalizing problems. An explanation for the predominance of internalizing problems is that these problems counteract against externalizing problems, meaning that adolescents who report high levels of anxious or depressed behavior may inherently avoid aggressive and rule-breaking behaviors. Our effect sizes for internalizing problem behavior are slightly higher than the average effect size of *d* = .23 in the field (Sieh et al. 2010a). While we found no significant effect for externalizing problem behavior, the meta-analysis from Sieh et al. (2010a) reported a significant average effect size of *d* = .09 with several studies showing a marked group difference (Hough et al. 2003; Tompkins and Wyatt 2008). A possible explanation is that studies with medium to large effects for externalizing problems focused on a different diagnostic group. Those studies were non-cancer studies and mainly included children of parents with HIV in families characterized by a low socio-economic status. Accordingly, studies showing that externalizing problems were less prevalent in the target group than in the comparison group analyzed a mixed illness sample (Barkmann et al. 2007) or cancer in families with high socio-economic status (Visser et al. 2007).

With respect to caregiving variables, the effect sizes were medium except for caregiving responsibilities, demonstrating the importance of taking outcomes into consideration that can be a specific result of the impact of parental illness. Specifically, the target group appears to be characterized by activity restrictions, feeling of isolation and high frequency of household chores. Our effect sizes for activity restrictions and feeling of isolation were medium like in the study of Pakenham et al. (2006). On the contrary, our effect size for caregiving responsibilities was small versus an effect size of *d* = .70 in the study from Pakenham et al. This discrepancy is probably due to sample differences, that is, Pakenham et al. examined caregivers versus non-caregivers. Self-identified caregivers may inherently score higher on caregiving responsibilities.

Moreover, the target group comparatively experienced more daily hassles that negatively affected their personal life during leisure time and school duties. A study found that a higher frequency of daily hassles predicted problem behavior (Meijer et al. 2008), so daily hassles may be a crucial risk factor for problem behavior in the target group. Similarly, the stress level in the target group was pronounced, which is in line with prior research (Houck et al. 2007; Huizinga et al. 2005b; Verhaeghe et al. 2005).

A positive outcome for the target group was that coping skills seemed to be fairly equal between the two groups. This is in contrast to what was expected based on prior research (Pakenham and Bursnall 2006). It is possible that we did not find a significant effect because we used a questionnaire that may be less sensitive to detect caregiving- or illness-related coping. Nonetheless, the Utrecht Coping List is a valid and reliable measure for active problem solving and social support seeking, and our results clearly indicate that the target group exhibited these skills to the same degree as adolescents who were not affected by parental CMC. The quality of parent attachment did not differ between the groups either. This is a valuable finding in the sense that children of parents with CMC could fall back on their parents for support to the same degree as controls. All the same, it may be argued that children need extra coping skills and require highly secure parent attachment to buffer the risk for adjustment difficulties.

Furthermore, the target group reported worse grades and failed a school year slightly more often than teenagers from the comparison group. Hedges and Hedberg (2007) affirm that a small effect size of *d* = .20 for academic achievement is of policy interest. In our study, the effect size for GPA was medium, which can be regarded as a large difference in the area of academic achievement. Banks et al. (2002) concluded that adolescents have educational difficulties as a result of caring. Another explanation is that lower academic achievement is mediated by elevated levels of anxiety which are more common in the target group, so especially children with preoccupations and anxieties concerning their parent’s illness perform worse at school (Duchesne and Ratelle 2010). Noteworthy, academic achievement is the main route into unemployment and adolescents with a chronically ill parent should not be at a disadvantage (Dearden and Becker 2000). The target group followed intermediate vocational education decisively more often and high school less often than controls, while there was no group difference between parents’ educational level. Davis-Kean (2005) affirmed that parents’ and children’s educational level highly correlate, suggesting that parents’ expectations influence children’s academic choices. In our study, no such correlation was found, so it can be assumed that adolescents with a chronically ill parent are rather influenced by their personal needs or by economic needs of the family than by parents’ education.

This study had some limitations. First, the sample was select considering that the ethnic composition was largely Caucasian, which may limit the generalizability of our results. In addition, only CMC’s combined with impairments were included and some conditions were excluded, for example, cancer and HIV. Consequently, it should be avoided to make general statements about children of specific illness groups. Second, we exclusively examined group differences, hence, no statements can be made about predictors for adjustment difficulties and generally, no causal relationships can be derived from growing up with a parent who has been diagnosed with CMC. Third, the large amount of variables investigated could have resulted in type 1 errors, meaning that the chance to find significant effects augments with the number of variables. Several effect sizes are medium and implicate a lower chance of type 1 errors, while other effect sizes are lower than Cohen’s *d* = .3 and should be interpreted with more caution. Notwithstanding, Durlak (2009) argues that even small effect sizes can be considerate depending on the research area. In addition, we believe that the chance for type 1 errors is unlikely because we found significant effects for the majority of variables. Besides, we categorized several variables within domains (e.g., caregiving) and found effects for most domains in consistence with the literature that demonstrates that parental illness has impact on children’s caregiving outcomes and internalizing problems (e.g., Barkmann et al. 2007; Pakenham et al. 2006; Sieh et al. 2010a). Fourth, our sample had a wide age range. This may be an issue because the developmental variability could have played a large role, so differences within certain age groups could be smaller or larger than what we found for the whole sample. Thus, conclusions with respect to pubertal stages and narrow age groups cannot be drawn. Finally, we were not able to investigate which specific factors accounted for the variance explained by the family cluster effect. A follow-up study should also assess variables at the family level (e.g., family cohesion) to be entered into the analyses. Future research needs to replicate this study with a culturally diverse sample or focus on non-western cultures. It should be noted that illness is not defined as a medical condition in some cultures. Parents with CMC from different cultures with similar symptoms may have distinct illness-related interpretations and coping systems, which in turn may affect child outcomes in different ways (Helman 2007).

In conclusion, this study shows that the family cluster effect explains a large proportion of adolescent outcomes and illustrates distinct features of adolescents with a chronically ill parent. The target group appears to display more internalizing problems compared to adolescents with two healthy parents. They seem to perceive adverse effects of caregiving and experience daily hassles and stress. In addition, their GPA is comparatively low. Hence, growing up with a chronically ill parent appears to pose a risk for behavioral, psychosocial and academic problems of adolescents. Professionals and teachers dealing with children should consider the possibility of parental illness and accordingly, be alert to signs of fears, depressed mood, somatic complaints, isolation and academic underachievement. In congruence with a family-centered approach (Gorter et al. 2010), we recommend professionals to communicate with parents and children about the diagnosis and the short and long term impact of parental illness on the family. Parents should be encouraged to make sure that their children have enough illness-related information and know how to deal with the medical condition. Considering that clinical levels of stress poses a threat to a substantial part of the target group (Houck et al. 2007), clinicians and researchers should collaborate to create evidence-based interventions aiming to reduce stress in this specific group. An intervention that may specifically help the target group is stress management which particularly makes use of strengths and important resources such as coping skills (Murray and Pizzorno 2006). Negative consequences of caregiving responsibilities and illness demands on child development may be a major concern. In this respect, it should be evaluated whether children need assistance for caregiving tasks, leaving sufficient time for their leisure and school activities.
